# The utility of MRI radiological biomarkers in determining intracranial pressure

**DOI:** 10.1038/s41598-024-73750-9

**Published:** 2024-10-05

**Authors:** Anand S. Pandit, Musa China, Raunak Jain, Arif H. B. Jalal, Maria Jelen, Shivani B. Joshi, Crystallynn Skye, Zakee Abdi, Yousif Aldabbagh, Mohammad Alradhawi, Ptolemy D. W. Banks, Martyna K. Stasiak, Emily B. C. Tan, Fleur C. Yildirim, James K. Ruffle, Linda D’Antona, Hasan Asif, Lewis Thorne, Laurence D. Watkins, Parashkev Nachev, Ahmed K. Toma

**Affiliations:** 1 High-Dimensional Neurology Group, UCL Queen Square Institute of Neurology, University College London, London, UK; 2 Victor Horsley Department of Neurosurgery, National Hospital for Neurology and Neurosurgery, London, UK; 3 Division of Medicine, University College London, UCL, London, UK; 4 Department of Psychology and Language Sciences, University College London, London, UK; 5 School of Medicine, Barts and the London School of Medicine and Dentistry, Queen Mary University of London, London, UK

**Keywords:** Adult hydrocephalus, Bayesian modelling, Radiological biomarkers, Predictive medicine, Neurological disorders, Computational biology and bioinformatics, Image processing

## Abstract

Intracranial pressure (ICP) is a physiological parameter that conventionally requires invasive monitoring for accurate measurement. Utilising multivariate predictive models, we sought to evaluate the utility of non-invasive, widely accessible MRI biomarkers in predicting ICP and their reversibility following cerebrospinal fluid (CSF) diversion. The retrospective study included 325 adult patients with suspected CSF dynamic disorders who underwent brain MRI scans within three months of elective 24-h ICP monitoring. Five MRI biomarkers were assessed: Yuh sella grade, optic nerve vertical tortuosity (VT), optic nerve sheath distension, posterior globe flattening and optic disc protrusion (ODP). The association between individual biomarkers and 24-h ICP was examined and reversibility of each following CSF diversion was assessed. Multivariate models incorporating these radiological biomarkers were utilised to predict 24-h median intracranial pressure. All five biomarkers were significantly associated with median 24-h ICP (*p* < 0.0001). Using a pair-wise approach, the presence of each abnormal biomarker was significantly associated with higher median 24-h ICP (*p* < 0.0001). On multivariate analysis, ICP was significantly and positively associated with Yuh sella grade (*p* < 0.0001), VT (*p* < 0.0001) and ODP (*p* = 0.003), after accounting for age and suspected diagnosis. The Bayesian multiple linear regression model predicted 24-h median ICP with a mean absolute error of 2.71 mmHg. Following CSF diversion, we found pituitary sella grade to show significant pairwise reversibility (*p* < 0.001). ICP was predicted with clinically useful precision utilising a compact Bayesian model, offering an easily interpretable tool using non-invasive MRI data. Brain MRI biomarkers are anticipated to play a more significant role in the screening, triaging, and referral of patients with suspected CSF dynamic disorders.

## Introduction

Abnormal intracranial pressure (ICP) represents an important pathophysiological parameter that portends neurological harm. Raised ICP can manifest as an acute phenomenon in patients following trauma and in those with intracranial space-occupying lesions. It is also apparent in patients whose disease process is more insidious and without an underlying structural cause as in the case of idiopathic intracranial hypertension (IIH)^1,2^.

Invasive ICP measurement via intraparenchymal monitoring or external ventricular drain remains the gold standard method of continuous measurement of ICP^2,3^. Whilst direct monitoring tools provide accurate, continuous real-time measurements of ICP, their invasive nature carries surgical risks and requirements for inpatient hospitalisation: limiting their use to centres with a neurosurgical service. Alternatively, lumbar puncture and manometry provide a ‘snapshot’ estimate of ICP that is susceptible to inaccuracy, dependent on patient positioning, physiological and diurnal variations^4,5^ and carries procedural risk.

Much desired are non-invasive assessments of ICP that can guide the management of patients with suspected, dynamic cerebrospinal fluid (CSF). Ophthalmological markers such as changes to the optic disc, lack of spontaneous venous pulsation and ultrasound markers of an enlarged optic nerve sheath show promise in predicting raised ICP^6,7^. However, there are practical considerations regarding availability and training, and the sensitivity of these markers remains unclear^8^.

Brain MRIs are widely accessible in non-emergency settings and are commonly performed in elective patients with suspected raised ICP. In addition to pituitary gland shape, MRI biomarkers such as distension of the optic nerve sheath, flattening of the posterior globe, tortuosity and protrusion of the optic nerve have been posited as proxy indicators of raised ICP in patients with IIH^9–15^. However, few studies correlate these imaging features with ICP measurements, nor have been examined in patient cohorts with sizable numbers or with CSF problems other than IIH. Others have utilised these biomarkers to predict ICP, dividing patient cohorts in a dichotomous manner as per arbitrary thresholds for raised ICP that may vary across different patient cohorts^15^.

To that end, we evaluate the utility of common MR-imaging biomarkers in prediction of intracranial pressure in the largest cohort of neurosurgical patients admitted for 24-h intracranial pressure recording available. Each biomarker is assessed individually and within classical and Bayesian multivariate models which can provide an estimate of predictive certainty. As a secondary aim, in patients who have had CSF diversion and attempted normalisation of ICP, we determine whether these biomarkers are reversible.

### Methods

## Ethics and guidelines

This use of data from this cohort was approved by the regional Research Ethics Committee—North East-Newcastle & North Tyneside 2 Research Ethics Committee and the Health Research Authority (20/NE/0127) who waived written informed consent due to its retrospective nature. All patients were informed and consented to ICP monitoring. The methods were performed in accordance with relevant guidelines, regulations and the TRIPOD checklist for multivariate predictive models.

### Patients

A retrospective review of our institutional database was conducted to identify a consecutive series of all adult patients investigated with elective 24-h intraparenchymal ICP monitoring between January 2006 and December 2021 at a large tertiary neurosciences centre. Patients meeting the following eligibility criteria were selected: (1) they had undergone elective 24-h ICP monitoring for a suspected CSF dynamic disorder and (2) had a brain MRI performed within three months of ICP monitoring, following a protocol previously described^15^. Patients who had CSF diversion prior to the monitoring were also included. Unless otherwise specified, all patient diagnoses were made by a consultant neurologist or neurosurgeon following evaluation of the combined clinical, imaging and ophthalmological information. For reversibility analysis, all patients who had a brain MRI following CSF diversion were included. Excluded were patients whose imaging was significantly affected by artefact rendering biomarker interpretation impossible or had insufficient or low quality ICP monitoring data.

### ICP monitoring

Within our institution, patients with suspected CSF dynamics abnormalities undergo elective ICP monitoring after discussion in a multidisciplinary team meeting consisting of neurosurgery and neurology attendings with a specialist interest in hydrocephalus and CSF disorders. A detailed description of the standardised protocol for the elective 24-h ICP monitoring procedure at our institution has previously been published^16^. Our standard operating procedure involves insertion of a right frontal Raumedic™ intraparenchymal ICP probe under local anaesthesia with or without sedation in the operating theatre and the patient is monitored for a continuous period of 24 h (Supplementary Methods).

### Neuroradiology

Diagnostic brain MRI scans performed within three months of the elective 24-h ICP monitoring were selected. Although the majority were performed internally at our centre, external transferred scans were used if these were not available. Although protocols and specific acquisition parameters varied by clinical indication and location for MR-imaging, all the patients had T1- or T2-weighted scans performed with a mean slice thickness of 5 mm or less as is the current neurological standard. For internally acquired scans, this was performed on Siemens Magnetom Skyra or Prisma scanner at 3 T with turbo spin echo protocol for the T2-weighted sequence.

**Fig. 1:**
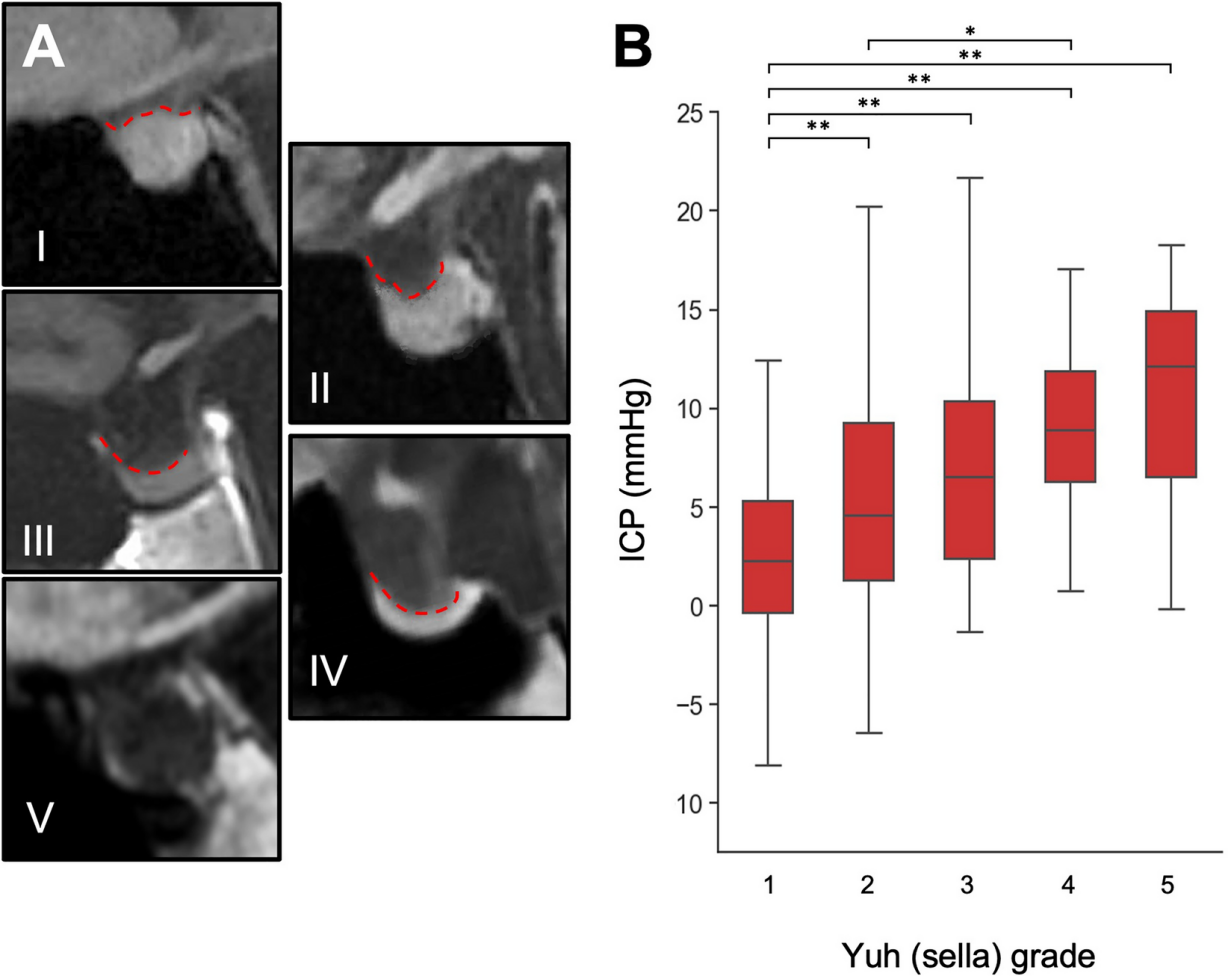
Yuh sella grading in selected patient examples using T1-weighted sagittal images (**A**), and association with 24-h median ICP (**B**). (*p < 0.05; **p < 0.01; ***p < 0.001).

**Fig. 2:**
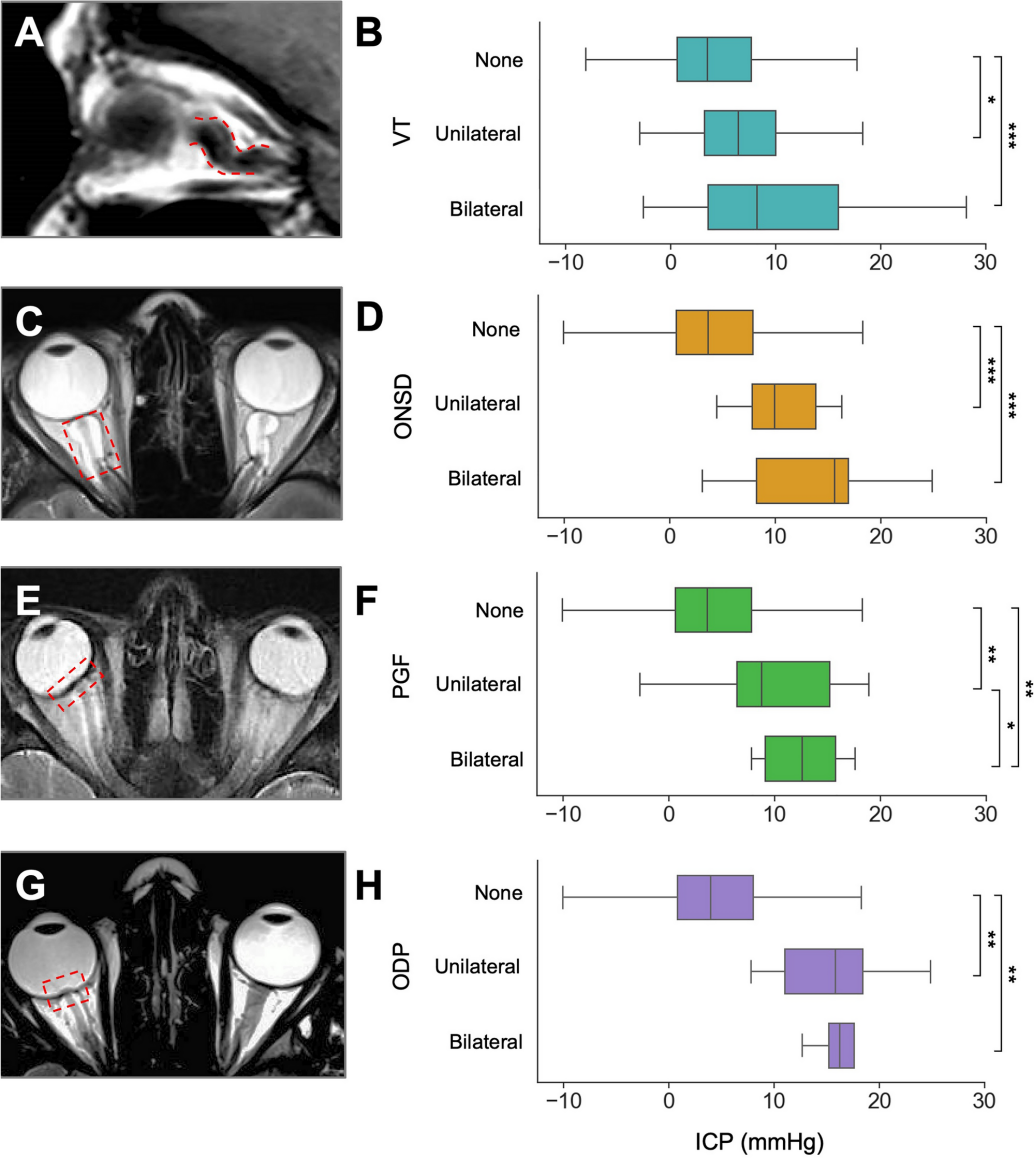
Non-pituitary MRI markers in selected patient examples using sagittal T1-weighted or axial T2-weighted imaging and associated 24-h median ICP measurements for optic nerve vertical tortuosity (**A**,** B**); optic nerve sheath distension (**C**,** D**); posterior globe flattening (**E**,** F**); optic disc protrusion (**G**,** H**).

Pituitary height and optic nerve characteristics were assessed using T1-weighted sagittal and T2-weighted axial brain MRI sequences. Maximum pituitary gland height was measured on mid-sagittal T1w scans and graded according to the Yuh classification^12^, with grades 3–5 considered abnormal (Fig. 1A). Optic nerve vertical tortuosity (VT) was confirmed by the "s-shaped" appearance on T1w sagittal sequence MRI (Fig. 2)^9^. Optic nerve sheath distension (ONSD), optic disc protrusion (ODP), and posterior globe flattening (PGF) were graded using non-fat-suppressed T2-weighted axial brain MRI sequences (Fig. 2). ONSD was confirmed when the hyper-intense CSF-containing sheath around the optic nerve measured more than 2 mm in width at any point up to 10 mm behind the globe^9^. ODP was confirmed by intraocular protrusion of the optic disc and associated scleral concavity^9,14^. Grades 0, 1 and 2 were assigned indicating none, one, or both eyes affected, with abnormal grading if at least one optic nerve was impacted. Biomarker assessment was performed in several stages in order to assure inter-rater reliability and was independently graded by a board-certified neuroradiologist blinded to clinical and ICP information with moderate to high inter-rater reliability (Supplementary Methods).

### Data analysis

All statistical analyses were performed in Python (v = 3.8.2). Univariate testing examined the association between individual biomarkers and median 24-h ICP. Group averages were compared using either a two-sided independent samples t-test or Mann–Whitney U (MWU) and Kruskal–Wallis (KW) tests depending on whether the data was parametric. Normality of data was assessed by histogram visualisation and using the Kolmogorov–Smirnov test. Both raw and binarised (normal/abnormal) grading were evaluated. Significant results were reported if *p* was < 0.05 following multiple comparison correction using the Holm-Bonferroni method. Reversibility testing of each radiological biomarker was assessed using either a paired t-test or Wilcoxon-Sign-Rank test in patients who were surgically-naive and then underwent CSF diversion.

Multivariate testing was first performed using an ordinary least squares (OLS) regression examining the association between radiological biomarkers and ICP, with and without adjustment for clinical and demographic variables. Here, model assumptions were verified including assessment of homoscedasticity and normality among the residuals. Next, a Bayesian multiple linear regression model was fitted which has several benefits relative to classical methods^17^ (Supplementary Methods). Overall model performance was evaluated using standardised scores: R^2^ and mean absolute error based on leave-one-out cross validation^18^.

Study size was determined pragmatically using all the available data. However, post-hoc analysis using the weakest model (OLS radiological, multivariate) with an adjusted R^2^ of 0.26 would yield a Cohen f^2^ effect size of 0.35. With an alpha error of 0.05, and given the number of subjects in the model, this would yield a power of 1 (GPower, v = 3.1).

## Results

### Patient characteristics

325 patients (229 female) met our participant criteria with a mean age of 40 years (SD = 15.1) (Table 1). The most frequent diagnosis or suspected diagnosis prior to ICP monitoring was IIH (30%), followed by patients with syringomyelia or a Chiari malformation (19%). 65 patients (20%) had no formal diagnosis or underlying structural abnormality and were being investigated for persistent, non-specific high or low intracranial pressure symptoms. The mean average of the 24-h ICP across the cohort was 5.41 (± 6.77 mmHg). The median time between ICP monitoring and MRI scan was 31 days. No correlation was found between median ICP and the MRI time interval. 144 patients had a prior CSF-diverting intervention, of which the majority had insertion of a ventriculoperitoneal shunt. No significant difference was found in average ICP between patients with (5.25 mmHg) and without prior CSF diversion (5.53 mmHg).

**Table 1:** Summary of patient demographics, underlying conditions and interventions.

Patients	325
Mean age (years)	40.1 + /- 15.1
Gender (F/M [%])	229/96 [70.5/29.5]
Primary pathology (%)	IIH = 97 (29.8) Chiari-Syringomyelia = 61 (18.8) Tumour / SOL = 32 (9.8) Congenital = 18 (5.5) NPH = 15 (4.6) Aqueductal Stenosis = 13 (4.0) LOVA = 10 (3.1) CSF leak = 8 (2.5) Neurovascular = 3 (0.9) Traumatic brain injury = 3 (0.9) Non-specific = 65 (20.0)
Median days between ICPM and MRI (IQR)	31 (7–60)
Prior intervention (%)	None = 181 (55.7) VPS = 88 (27.1) LPS = 17 (5.2) FMD = 6 (1.8) VSS = 5 (1.5) ETV = 3 (0.9) CFO = 3 (0.9) VAS = 2 (0.6) CPS = 2 (0.6) EVD = 2 (0.6) Multiple = 16 (4.9)
Surgical intervention following ICP / MRI (%)	No further surgical intervention = 239 (73.5) VPS = 59 (18.1) LPS = 6 (1.8) LD = 5 (1.5) FMD = 5 (1.5) CFO = 3 (0.9) VSS = 2 (0.6) EVD = 1 (0.3) VAS = 1 (0.3) SPS = 1 (0.3) Multiple = 3 (0.9)

*CFO* cyst fenestration with Ommaya reservoir insertion; *CPS* cysto-peritoneal shunt; *ETV* endoscopic third ventriculostomy; *EVD* external ventricular drain ;*FMD* foramen magnum decompression; *ICPM* intracranial pressure monitoring; *IIH* = idiopathic intracranial hypertension; *IQR* interquartile range; *LD* lumbar drain;*LOVA* long standing overt ventriculomegaly in adults; *LPS* lumboperitoneal shunt; *NPH* normal pressure hydrocephalus; *SOL* space-occupying lesion; *SPS* syringo-pleural shunt; *VAS* ventriculo-atrial shunt; *VPS* ventriculoperitoneal shunt; *VSS* venous sinus stenting.

## The association of individual radiological biomarkers with intracranial pressure

### Sella grade

A higher intracranial pressure was associated with an emptier sella, showing a stepwise increase in ICP between each grade (Kruskal–Wallis statistic = 57.2, *p* < 0.0001, Fig. 1B). Significant differences were observed between sella grade I and all other grades, as well as between sella grade II and IV. Abnormal sella morphology (Yuh grade 3–5) was linked with higher ICP (9.5 mmHg) compared to those with ‘normal’ sella shape (Yuh grades 1–2, 3.5 mmHg) [Mann–Whitney U = 8596, *p* < 0.0001].

### Vertical tortuosity

Patients with tortuous optic nerves had a higher intracranial pressure (KW = 17.2, *p* < 0.001), with a significant pairwise increase between none and unilateral and none and bilateral signs (Fig. 2A, B). Those with either one or two tortuous optic nerves had significantly higher median ICP (6.7 mmHg) as compared to those without signs of tortuosity (3.5 mmHg, MWU = 12,358, *p* < 0.0001).

### Optic nerve sheath distension

Patients with a distended optic nerve sheath had a higher intracranial pressure (KW = 29.4, *p* < 0.0001), with a stepwise increase between those with no ONSD and unilateral ONSD or bilateral ONSD (Fig. 2C, D). Those with one or two optic nerves exhibiting nerve sheath distension had significantly higher median ICP (10.1 mmHg) as compared to those without signs (3.7 mmHg, MWU = 5605.5, *p* < 0.0001).

### Globe flattening

Patients with optic globe flattening had a higher intracranial pressure (KW = 30.2, *p* < 0.0001), with a significant pairwise increase between none, unilateral and bilateral signs (Fig. 2E, F). Patients with one or two optic globes showing flattened appearances had significantly raised ICP (10.1 mmHg) as compared to those without signs (3.7 mmHg, MWU = 6839.5, *p* < 0.0001).

### Optic disc protrusion

Patients with protruded optic discs had a higher intracranial pressure (KW = 21.1, *p* < 0.0001), with a significant pairwise increase between none and unilateral and none and bilateral signs (Fig. 2G,H). Those with evidence of uni- or bilateral optic disc protrusion had significantly raised median ICP (16.2 mmHg) as compared to those without signs (4.0 mmHg, MWU = 3278, *p* < 0.0001).

After exclusion of patients who had surgical CSF diversion, the significance of the above results with were maintained (Supplementary Table 1), except for vertical tortuosity.

### Reversibility

36 patients (28 female) met the criteria for subgroup reversibility analysis with a mean age of 40 years (SD = 18.0). The most frequent diagnosis prior to ICP monitoring and intervention was a Chiari malformation (17%) followed by IIH (14%). Four patients (11%) had no formal diagnosis or underlying structural abnormality and were being investigated for persistent, non-specific high or low intracranial pressure symptoms. This smaller cohort was statistically equivalent to the remainder of the cohort with respect to age, sex and diagnosis. Here, the median interval between intervention and post-intervention MRI scan was 235 days (IQR: 74–529).

Among patients who were surgically naive and then who had CSF diverting treatment following ICP monitoring, only the sella grade was found to be reversible (pre-intervention Yuh median = 2 (IQR: 1–3), post-intervention Yuh median = 1 (IQR: 1–2), Wilcoxon Sign-Rank, *p* < 0.001) (Supplementary Table 2). ONSD demonstrated a trend toward reversibility (*p* < 0.1), although this value was uncorrected.

### Multivariate prediction of ICP using radiological markers

Three multivariate models were fitted to best characterise the association between radiological markers and intracranial pressure. In all three models the use of multiplicative interaction terms showed no improvement in model scoring and are therefore reported without interaction.

The first model, a sparse ordinary least squares (OLS) model, simulated a clinician reviewing an MRI scan *without* additional clinical information. Pairwise Cramér's V associations addressed collinearity, leading to the removal of ONSD due to its strong correlation with PGF (Supplementary Fig. 1), resulting in no change in adjusted R^2^. This model, explaining 26% of the variance, demonstrated all four remaining radiological markers significantly and independently associated with ICP, outperforming univariate OLS regression (Table 2, Supplementary Table 3).

**Table 2:** Comparison of multivariate models used to predict 24-h median intracranial pressure.

Model	Radiological (univariate)	Radiological (multivariate)	Clinico-radiological	Clinico-radiological
Method	OLS	OLS	OLS	Bayesian linear
Adjusted R^2^	0.03—0.10	0.26	0.33	0.37
LOO-CV Mean Absolute Error (mmHg)	3.83—4.00	3.20	2.83	2.71

Variable	Coefficient (CI)	p	Coefficient (CI)	p	Coefficient (CI)	p	Mean coefficient (94% CrI)
*Clinical*							
Age					- 0.07 (-0.12 – -0.03)	0.003	-0.07 (-0.11 – -0.03)
Diagnosis					Variable*	-	Variable**
*Radiological*							
Yuh	2.25	< 0.0001	1.77 (1.19—2.36)	< 0.0001	1.76 (1.19—2.32)	< 0.0001	1.76 (1.19—2.28)
VT	2.74	< 0.0001	2.03 (1.04—3.03)	< 0.0001	1.96 (0.99—2.92)	< 0.0001	1.95 (1.05—2.88)
PGF	4.27	< 0.0001	1.74 (0.05—3.42)	0.04	0.69 (-0.95—2.33)	ns	0.70 (-0.83—2.32)
ODP	7.28	< 0.0001	4.57 (2.09—7.05)	< 0.0001	3.62 (1.22—6.03)	0.003	3.61 (1.33—5.89)
ONSD	4.69	< 0.0001					
Intercept	-	-	0.68 (-0.64—2.00)	ns	3.51 (-0.11—7.12)	0.06	3.49 (-0.03—6.94)

*CI* confidence interval; *CrI* credible interval; *LOO-CV* leave-one-out cross validation; *ns* non-significant; *ODP* optic disc protrusion, *OLS* ordinary least squares; *ONSD* optic nerve sheath diameter; *PGF* posterior globe flattening; *VT* vertical tortuosity; Yuh = pituitary sella grade. (*see Supplementary Table 2; **see Supplementary Table 3).

In the second, a more complete OLS model was fitted that included salient demographic and clinical variables including patient age, sex, suspected diagnosis and whether they had a previous surgical intervention for CSF diversion. Variables were randomly permuted and the model which minimised the Bayesian Information Criterion the most was selected (i.e., the most parsimonious). In this way, patient age and diagnosis were retained, and previous surgery was not found to be informative. This model explained around a third of the variance (adjusted R^2^ = 0.33). Here, sella grade, VT and ODP remained significant after clinical confounder adjustment (Table 2, Supplementary Table 4). Individual diagnoses were not significantly associated with ICP after multivariate adjustment; however, age did remain significant.

**Fig. 3:**
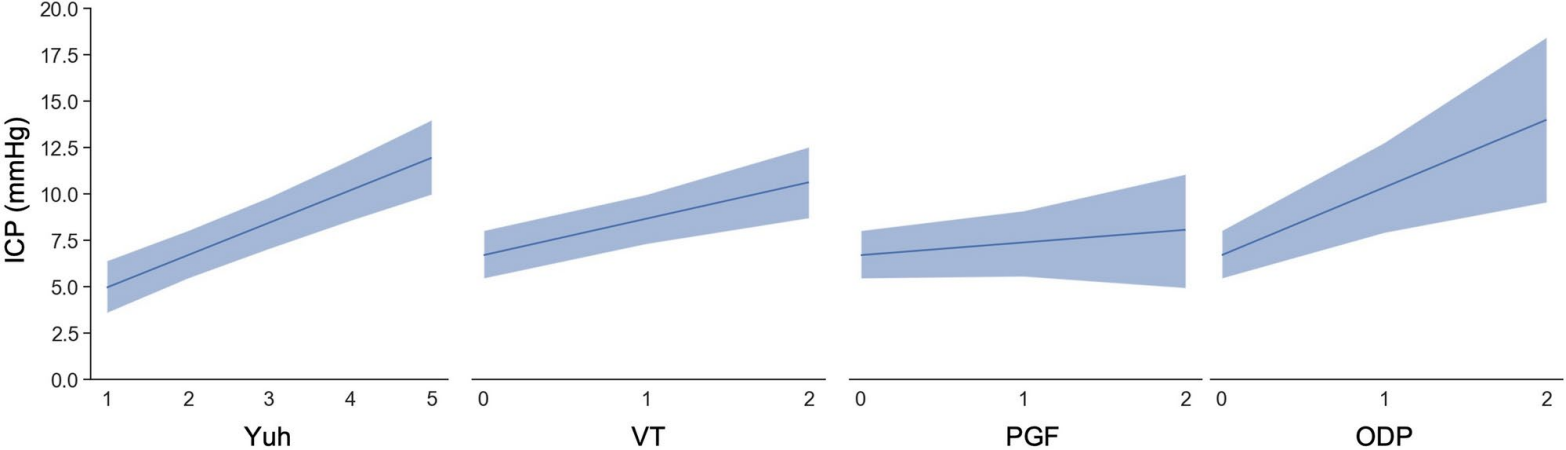
Conditional adjusted predictions for individual radiological biomarkers. All other covariates, including other radiological markers, are constant at their sample mean. Dark blue line represents mean with 94% credible intervals shown in the pale blue bars with interpolation. ODP = optic disc protrusion, PGF = posterior globe flattening, VT = vertical tortuosity, Yuh = pituitary sella grade.

In the third, a Bayesian linear model was fitted using the previous clinical and radiological variables, however here no specification was made on the relationship between independent variables and median ICP. This had a higher adjusted R^2^ (0.37) as compared to the previous model, a and a mean absolute error of 2.71 mmHg. Further, one could assess the degree of credibility in the coefficients of each imaging variable when other variables were held at the sample mean (Fig. 3).

## Discussion

### Summary of results

This observational study explores the correlation between conventional MRI biomarkers and 24-h median intracranial pressure (ICP) in 325 neurosurgical patients undergoing ICP monitoring. As anticipated, patients with radiological abnormalities exhibited significantly higher 24-h median ICP readings. Multivariate analyses, employing both frequentist and Bayesian approaches, revealed positive associations between Yuh sella grading, vertical tortuosity, optic disc protrusion, and ICP which were found to be independent of each other. The Bayesian multiple linear regression model exhibited superior predictive performance, with a mean absolute error of 2.71 mmHg, indicating potential clinical utility for triaging adult patients with ICP abnormalities. Following CSF diversion in surgically naive patients, it was found that pituitary deformation resolved, supporting the posited reversibility of sella imaging biomarkers.

### Interpretation of results and context

Accurate, non-invasive prediction of ICP remains a much-desired tool for selected patient cohorts with suspected CSF-disorders aiding in screening, triage and referral. While clinical signs associated with abnormal ICP have historically been well described, these lack specificity^19,20^ and ophthalmological and ultrasound-based markers, though valuable, may be limited in availability. In contrast, routinely obtained conventional MRI sequences provide a feasible means to assess ICP through morphological effects on optic nerve, globe, and sella. Previous MR imaging studies have been limited to small patient cohorts, typically with IIH and are often correlated with non-standardised ICP measurements^9,12,21–23^. Others divide patient cohorts dichotomously using arbitrary thresholds for raised ICP, which are variable across different patient cohorts^15,24^. Yet in spite of these differences, our univariate results remain broadly in tandem with the existing literature.

In a cohort of IIH patients, Rohr et al. found that a number of radiographic MR imaging signs were positively associated with lumbar puncture (LP) opening pressure, whereas Tuncel et al. found no such correlation^22,23^. Variations in observations may result from the differential weighting of imaging markers or the reliance on single LP measurements^25^. Using 24-h intraparenchymal ICP readings, D’Antona et al. investigated the association between pituitary gland shape, ONSD, VT, and ODP and median ICP within a cohort of 45 patients with a wide range of CSF dynamic disorders^15^. They found all four biomarkers, when abnormal, were significantly associated with raised 24-h ICP readings and in 94% of patients in whom all four radiological signs were absent ‘normal’ median ICP readings (< 5.96 mmHg) were exhibited. With the addition of PGF, we also found that abnormal radiographic markers were significantly associated with increased 24-h median ICP on univariate analyses and were associated with an even higher ICP if the marker was present bilaterally.

Our study suggests that the relationship between radiological variables and intracranial pressure (ICP) is complex. We used multivariate modelling methods to characterise this relationship more precisely. By using continuous ICP measurements, we eliminated binary assumptions about what defines raised ICP. We found that three markers (Yuh sella grade, VT, ODP) remained positively associated with ICP even after adjustment, and our model was able to predict median ICP readings with a clinically useful error. Furthermore, our study identified the pituitary gland’s responsiveness to attempted ICP normalization following CSF diversion, consistent with previous findings post-lumbar puncture^22^ or medical treatment^21^. While other radiological markers may exhibit reversibility with adequate power or extended intervals, caution is warranted in interpreting the clinical utility of this reversibility due to the limited sample size in the specific subgroup analysis. Additional work is necessary to confirm whether morphological resolution of intracranial structures is associated with clinical improvement.

### Limitations and strengths

We note several limitations in our work. Firstly, this was an observational study design that included mostly elective admissions for investigating sub-acute CSF dynamic disorders, making our findings less relevant to patients who present emergently or have other conditions. The distribution of median ICP was wide (Supplementary Fig. 2A), with less calibration for negative and extremely positive results (Supplementary Fig. 2B). Secondly, there was a latency period between MRI scan and ICP monitoring, in which there could be radiographic changes and reduce the reliability of our principal findings. However, when accounted for statistically, this variable did not affect the model fit or significance (Supplementary Results). Thirdly, other radiological variables such as venous sinus stenosis^22^ and other sequences^10,26^, were not recorded due to our institutional protocol. Isotropic image sequences with thin slice thicknesses could also improve the precision of the radiological grading. In addition, other outcome variables, such as intracranial pressure amplitude or frequency of prolonged elevated ICP episodes could have been of interest but were outside the scope of this work.

The strengths include a large and diagnostically diverse patient cohort, the use of both frequentist and Bayesian methods, and reliance on conventionally acquired MRI data facilitating robust and generalisable interpretation. The transparent and interpretable model enhances its accessibility to non-expert users.

To the best of our knowledge, this work offers several original contributions: (i) this is the first attempt to fit multivariate models for predicting intracranial pressure (ICP) using conventional neuroimaging. It also identifies which radiological markers, if any, have positive independent correlations with ICP; and (ii) we demonstrate a novel reversibility of certain imaging biomarkers following cerebrospinal fluid (CSF) diversion.

Predicting ICP will likely enhance clinical workflow by providing additional quantitative information to support or refute the need for shunting. It may assist in triaging and expediting patients for invasive ICP measurement when clinical information is uncertain. Further research is needed to assess the feasibility and optimal implementation of this type of algorithmic assistance in the patient care pathway.

## Conclusion

Our study establishes significant positive associations between three conventionally acquired MRI biomarkers (Yuh sella grading, VT, ODP) and 24-h median ICP after multivariate adjustment, thereby adding greater precision to their relevance. Both frequentist and Bayesian approaches support these findings, with a compact Bayesian multiple linear regression model offering potential practical clinical applicability. Future prospective studies would be strengthened by incorporating a standardised imaging protocol optimised for the assessment of ophthalmic and sella structures.

## Supplementary Information

Supplementary Information.

## Data Availability

The datasets generated during and/or analysed during the current study are not publicly available due to patient confidentiality but de-identified tabular data are available from the corresponding author (A.S.P) on reasonable request, provided ethical permissions have been obtained. All code used for analysis is available without undue reservation from the corresponding author.
